# Supplementary material for: Consensus based framework for digital mobility monitoring
Source: PLoS One. 2021 Aug 20;16(8):e0256541. doi: 10.1371/journal.pone.0256541 (PMC8378707; doi:10.1371/journal.pone.0256541)
Supplement: S1 File — (PDF) [file pone.0256541.s001.pdf]

S1 File

Questionnaire with initially proposed definitions of terms related to real-world walking assessed in round one.

Walking

Physiological

Human **walking** is a method of locomotion and is defined as initiating and maintaining a forward displacement of the centre of mass in an intended direction involving the use of the two legs, which provide both support and propulsion. The feet are repetitively and reciprocally lifted and set down whereby at least one foot is in contact with the ground at all times [1, 2]. **Walking** with walking aids is included in this definition.

A **step** is the interval between the initial contacts of the ipsi- and contralateral foot [1] and corresponds to the forward displacement of the foot together with a forward displacement of the trunk [3].

A **stride** is the interval between two successive initial contacts of the same foot. As such, a **stride** is equivalent to the gait cycle and every stride contains two **steps** [1].

	Strongly disagree	Somewhat disagree	No opinion	Somewhat agree	Strongly agree
Do you agree to the above definition?	☐	☐	☐	☐	☐

Relation to walking bouts

Walking is made up of walking bouts and is equivalent to taking steps/stepping forward (thus stepping in place does not constitute walking) and is defined as starting from initial contact for the initial step until ending with full floor contact of the foot making the last step [3].

	Strongly disagree	Somewhat disagree	No opinion	Somewhat agree	Strongly agree
Do you agree to the above definition?	☐	☐	☐	☐	☐

Please state any comments, further input or adapted definitions: ____________________

Purposeful

Characteristics

Purposeful walking occurs in discrete walking bouts and is therefore directly associated to the definition of a walking bout. Purposeful walking does not specify any gait characteristics (i.e. certain walking speed).

	Strongly disagree	Somewhat disagree	No opinion	Somewhat agree	Strongly agree
Do you agree to the above definition?	☐	☐	☐	☐	☐

Please state any comments, further input or adapted definitions: ____________________

Real-world

Characteristics

Real-world relates to the context in which walking takes place – that is free-living, unsupervised, uncontrolled and non-standardised. As such, it is unscripted as there are no instructions to the subject who does not need to interact with the wearable device(s).

Real-world actions occur in non-simulated everyday situations in unconstrained environments with minimal consciousness of being tested. It is equivalent to actions at home or in the community over continuous periods of time [4].

Synonymous terms are (environment of) **daily living**, or relating to **daily-life**. **Home environment** is used synonymously to **real-world** and **daily-life** without a separation of indoor and outdoor environment [5].

Real-world is distinct from laboratory-based [6], supervised (= fully controlled and observed), and semi-controlled (walking 'freely' but with supervision) tests. It also is different from scripted/instructed walking, which can take place in the home or lab (such as walking tests like the 4x10m test, 6-minute walk test (6MWT) and timed up and go (TUG)).

	Strongly disagree	Somewhat disagree	No opinion	Somewhat agree	Strongly agree
Do you agree to the above definition?	☐	☐	☐	☐	☐

Clinical environment

Free walking in hospitals is part of the **real-world** definition, but standardized supervised tests in a hospital are not. This excludes instructed actions, e.g. by medical professionals.

	Strongly disagree	Somewhat disagree	No opinion	Somewhat agree	Strongly agree
Do you agree to the above definition?	☐	☐	☐	☐	☐

Standardized measurement

Home-based tests, which are semi-standardized measurements performed in the home environment in a controlled or semi-controlled environment (such as short walk tests), are thus not regarded as being part of **real-world**. Home-based tests can nevertheless be an alternative to clinical tests and might be easier to conduct operationally and analyse than continuous monitoring (assuming standardized instructions).

	Strongly disagree	Somewhat disagree	No opinion	Somewhat agree	Strongly agree
Do you agree to the above definition?	☐	☐	☐	☐	☐

Please state any comments, further input or adapted definitions: ____________________

____

Walking bout

Characteristics

We define a **walking bout (WB)** as the sequence of walking periods that may be separated by breaks (resting periods). Each walking period is defined as an interval of at least three successive strides.

Note: We set no specific threshold for the maximum resting period between consecutive walking periods as the change of digital mobility outcomes with the resting period is unclear [7] and should be investigated for each clinical population separately.

	Strongly disagree	Somewhat disagree	No opinion	Somewhat agree	Strongly agree
Do you agree to the above definition?	☐	☐	☐	☐	☐

Please state any comments, further input or adapted definitions: ____________________

____

Walking speed

Physical definition

Walking speed (WS) is the distance covered by the whole body within a certain time interval / per unit time of walking. It is measured in meters per second and is the magnitude of the velocity vector (velocity includes direction and magnitude of walking) [2].

	Strongly disagree	Somewhat disagree	No opinion	Somewhat agree	Strongly agree
Do you agree to the above definition?	☐	☐	☐	☐	☐

Granularity

Walking speed can be estimated at different granularities:

- Instantaneous WS varies from one instant to another during the walking cycle [2]
- Step-wise WS is the ratio between step distance (length) and step time [4]
- Stride-wise WS [8]
- Averaged over WBs
- Averaged over other time intervals (hourly, daily, weekly) based on multiple WBs

The granularity by which the WS is assessed should be related to clinical parameters for each population separately.

	Strongly disagree	Somewhat disagree	No opinion	Somewhat agree	Strongly agree
Do you agree to the above definition?	☐	☐	☐	☐	☐

Relation to walking bouts

We assume that the minimal length of one walking period required to assess average **walking speed** for all clinical populations is 10 steps (5 strides).

	Strongly disagree	Somewhat disagree	No opinion	Somewhat agree	Strongly agree
Do you agree to the above definition?	☐	☐	☐	☐	☐

Please state any comments, further input or adapted definitions: ____________________

Turning

Characteristics

The process of **turning** consists of decelerating the forward motion, rotating the body as a whole, and stepping out toward the new direction [9]. Thus, **turning** includes a change of walking direction and change in angular orientation including a rotational movement of the body around the longitudinal axis. **Turning**, curvilinear walking, and straight walking involve different neuromotor strategies and need to be discriminated.

	Strongly disagree	Somewhat disagree	No opinion	Somewhat agree	Strongly agree
Do you agree to the above definition?	☐	☐	☐	☐	☐

Please state any comments, further input or adapted definitions: ____________________

References

1. Perry J. Ganganalyse: Norm und Pathologie des Gehens. 1st ed. München: Urban & Fischer; 2003.
2. Whittle MW. Gait analysis: An introduction. 3rd ed. Edinburgh: Butterworth-Heinemann; 2005.
3. Dijkstra B, Kamsma YP, Zijlstra W. Detection of Gait and Postures Using a Miniaturized Triaxial Accelerometer-Based System: Accuracy in Patients With Mild to Moderate Parkinson's Disease. Archives of Physical Medicine and Rehabilitation. 2010;91(8):1272–1277. doi:10.1016/j.apmr.2010.05.004.
4. Del Din S, Godfrey A, Galna B, Lord S, Rochester L. Free-living gait characteristics in ageing and Parkinson's disease: impact of environment and ambulatory bout length. Journal of NeuroEngineering and Rehabilitation. 2016;13(1):46. doi:10.1186/s12984-016-0154-5.
5. Brodie MA, Coppens MJ, Ejupi A, Gschwind YJ, Annegarn J, Schoene D, et al. Comparison between clinical gait and daily-life gait assessments of fall risk in older people. Geriatrics & Gerontology International. 2017;17(11):2274–2282. doi:10.1111/ggi.12979.
6. Iluz T, Gazit E, Herman T, Sprecher E, Brozgol M, Giladi N, et al. Automated detection of missteps during community ambulation in patients with Parkinson's disease: a new approach for quantifying fall risk in the community setting. Journal of NeuroEngineering and Rehabilitation. 2014;11(1):48. doi:10.1186/1743-0003-11-48.
7. Barry G, Galna B, Lord S, Rochester L, Godfrey A. Defining ambulatory bouts in free-living activity: Impact of brief stationary periods on bout metrics. Gait & Posture. 2015;42(4):594–597. doi:10.1016/j.gaitpost.2015.07.062.
8. Aminian K, Najafi B, Büla C, Leyvraz PF, Robert P. Spatio-temporal parameters of gait measured by an ambulatory system using miniature gyroscopes. Journal of Biomechanics. 2002;35(5):689–699. doi:10.1016/S0021-9290(02)00008-8.
9. Hase K, Stein RB. Turning Strategies During Human Walking. Journal of Neurophysiology. 1999;81(6):2914–2922. doi:10.1152/jn.1999.81.6.2914.
