# Supplementary material for: Consensus based framework for digital mobility monitoring
Source: PLoS One. 2021 Aug 20;16(8):e0256541. doi: 10.1371/journal.pone.0256541 (PMC8378707; doi:10.1371/journal.pone.0256541)
Supplement: Attachment — Submitted filename: Response_Reviewers.docx [file pone.0256541.s002.docx]

**Journal Paper Review**

**Journal:**	PLOS ONE

**Manuscript ID:**	PONE-D-21-01788

**Title:**	Consensus based framework for digital mobility monitoring

Dear Mr. Dobkin,

Thank you for reviewing and handling our manuscript. We addressed all the remaining requirements and included them into our manuscript (as indicated below).

Yours sincerely,

Felix Kluge

**Comments**

1. Please ensure that your manuscript meets PLOS ONE's style requirements, including those for file naming. The PLOS ONE style templates can be found at

<https://journals.plos.org/plosone/s/file?id=wjVg/PLOSOne_formatting_sample_main_body.pdf> and

[https://journals.plos.org/plosone/s/file?id=ba62/PLOSOne_formatting_sample_title_authors_affiliations.pdf](about:blank)

Response: We updated the formatting accordingly, especially regarding the authors page and tables.

2.Thank you for stating the following in the Competing Interests section:

"The authors have declared that no competing interests exist."

We note that one or more of the authors are employed by a commercial company: Novartis Pharma AG

a) Please provide an amended Funding Statement declaring this commercial affiliation, as well as a statement regarding the Role of Funders in your study. If the funding organization did not play a role in the study design, data collection and analysis, decision to publish, or preparation of the manuscript and only provided financial support in the form of authors' salaries and/or research materials, please review your statements relating to the author contributions, and ensure you have specifically and accurately indicated the role(s) that these authors had in your study. You can update author roles in the Author Contributions section of the online submission form.

Please also include the following statement within your amended Funding Statement.

“The funder provided support in the form of salaries for authors [insert relevant initials], but did not have any additional role in the study design, data collection and analysis, decision to publish, or preparation of the manuscript. The specific roles of these authors are articulated in the ‘author contributions’ section.”

If your commercial affiliation did play a role in your study, please state and explain this role within your updated Funding Statement.

Response: We updated the Funding Statement accordingly:

“All authors are part of the MOBILISE-D project. The MOBILISE-D project has received funding from the Innovative Medicines Initiative 2 Joint Undertaking under grant agreement No. 820820. This Joint Undertaking receives support from the European Union’s Horizon 2020 research and innovation program and the European Federation of Pharmaceutical Industries and Associations (EFPIA).

SDD and LR are supported by the National Institute for Health Research (NIHR) Newcastle Biomedical Research Centre (BRC) based at Newcastle Upon Tyne Hospital NHS Foundation Trust and Newcastle University. The work was also supported by the NIHR/Wellcome Trust Clinical Research Facility (CRF) infrastructure at Newcastle upon Tyne Hospitals NHS Foundation Trust.

AM is employed by the Novartis Pharma AG. The funder provided support in the form of salary for AM, but did not have any additional role in the study design, data collection and analysis, decision to publish, or preparation of the manuscript.

BME gratefully acknowledges the support of the German Research Foundation (DFG) within the framework of the Heisenberg professorship program (grant number ES 434/8-1).

CM is supported by the National Institute for Health Research (NIHR) Sheffield Biomedical Research Centre (BRC) based at Sheffield Teaching Hospital NHS Foundation Trust and University of Sheffield.

All opinions are those of the authors and not the funders. The funders had no role in study design, data collection and analysis, decision to publish, or preparation of the manuscript. The specific roles of the authors are articulated in the ‘author contributions’ section.”

b) Please also provide an updated Competing Interests Statement declaring this commercial affiliation along with any other relevant declarations relating to employment, consultancy, patents, products in development, or marketed products, etc.

Within your Competing Interests Statement, please confirm that this commercial affiliation does not alter your adherence to all PLOS ONE policies on sharing data and materials by including the following statement: "This does not alter our adherence to  PLOS ONE policies on sharing data and materials.” (as detailed online in our guide for authors [http://journals.plos.org/plosone/s/competing-interests](about:blank)) . If this adherence statement is not accurate and there are restrictions on sharing of data and/or materials, please state these. Please note that we cannot proceed with consideration of your article until this information has been declared.

Response: We updated the Competing Interests Statement accordingly:

“The authors have declared that no competing interests exist. The funders did not have any role in the study design, data collection and analysis, decision to publish, or preparation of the manuscript. The commercial affiliation does not alter our adherence to PLOS ONE policies on sharing data and materials.”

Please include both an updated Funding Statement and Competing Interests Statement in your cover letter. We will change the online submission form on your behalf.

Response: We included both statements in the cover letter.

Please know it is PLOS ONE policy for corresponding authors to declare, on behalf of all authors, all potential competing interests for the purposes of transparency. PLOS defines a competing interest as anything that interferes with, or could reasonably be perceived as interfering with, the full and objective presentation, peer review, editorial decision-making, or publication of research or non-research articles submitted to one of the journals. Competing interests can be financial or non-financial, professional, or personal. Competing interests can arise in relationship to an organization or another person. Please follow this link to our website for more details on competing interests: [http://journals.plos.org/plosone/s/competing-interests](about:blank)

3. One of the noted authors is a group or consortium [Mobilise-D consortium]. In addition to naming the author group, please list the individual authors and affiliations within this group in the acknowledgments section of your manuscript. Please also indicate clearly a lead author for this group along with a contact email address.

Response: We added the Acknowledgements section mentioning the specific individuals with affiliations. As Lynn Rochester being the academic lead of this consortium is already represented in the paper as co-author, we did not indicate an additional lead author of the consortium in the Acknowledgements section.

4. Please include additional information regarding the survey or questionnaire used in the study and ensure that you have provided sufficient details that others could replicate the analyses. For instance, if you developed a questionnaire as part of this study and it is not under a copyright more restrictive than CC-BY, please include a copy, in both the original language and English, as Supporting Information.

Response: We included the full questionnaire as supporting information and removed the S1 Table (Initial definitions of terms related to real-world walking assessed in round one), respectively.

5. Please provide additional details regarding participant consent. In the ethics statement in the Methods and online submission information, please ensure that you have specified what type you obtained (for instance, written or verbal, and if verbal, how it was documented and witnessed). If your study included minors, state whether you obtained consent from parents or guardians. If the need for consent was waived by the ethics committee, please include this information.

Response: We updated the ethics statement, as well as the Methods section:
“Ethical approval for this study was granted by the ethics committee of the University Hospital Erlangen (Re.-No. 241_19 Bc). All participants provided written informed consent before inclusion in the study. Participation in this study was voluntary. All data were handled in accordance with European data protection regulations.”
