# Supplementary material for: Consensus based framework for digital mobility monitoring
Source: PLoS One. 2021 Aug 20;16(8):e0256541. doi: 10.1371/journal.pone.0256541 (PMC8378707; doi:10.1371/journal.pone.0256541)
Supplement: Attachment — Submitted filename: Response_Reviewers.docx [file pone.0256541.s003.docx]

**Journal Paper Review**

**Journal:**	PLOS ONE

**Manuscript ID:**	PONE-D-21-01788

**Title:**	Consensus based framework for digital mobility monitoring

Dear Mr. Sakakibara, dear reviewers,

Thank you for reviewing and handling our manuscript. We addressed all the remaining requirements and included them into our manuscript (as indicated below).

Yours sincerely,

Felix Kluge

**Comments**

**Comments to the Author**

2. Is the manuscript technically sound, and do the data support the conclusions?

The manuscript must describe a technically sound piece of scientific research with data that supports the conclusions. Experiments must have been conducted rigorously, with appropriate controls, replication, and sample sizes. The conclusions must be drawn appropriately based on the data presented.

Reviewer #1: Yes

Reviewer #2: No

Response: Reviewer comments are handled as outlined below.

3. Has the statistical analysis been performed appropriately and rigorously?

Reviewer #1: Yes

Reviewer #2: No

Response: Reviewer comments are handled as outlined below.

4. Have the authors made all data underlying the findings in their manuscript fully available?

The PLOS Data policy requires authors to make all data underlying the findings described in their manuscript fully available without restriction, with rare exception (please refer to the Data Availability Statement in the manuscript PDF file). The data should be provided as part of the manuscript or its supporting information, or deposited to a public repository. For example, in addition to summary statistics, the data points behind means, medians and variance measures should be available. If there are restrictions on publicly sharing data—e.g. participant privacy or use of data from a third party—those must be specified.

Reviewer #1: Yes

Reviewer #2: No

Response: All data is fully available on <https://doi.org/10.5281/zenodo.4316564>, as stated in the Methods section. Also, the analysis code is available on <https://doi.org/10.5281/zenodo.4316739> .

5. Is the manuscript presented in an intelligible fashion and written in standard English?

PLOS ONE does not copyedit accepted manuscripts, so the language in submitted articles must be clear, correct, and unambiguous. Any typographical or grammatical errors should be corrected at revision, so please note any specific errors here.

Reviewer #1: Yes

Reviewer #2: Yes

Response: Thank you! Nevertheless, we included a revision by a native English speaker to eliminate potential language errors.

**Review Comments to the Author**

**Reviewer #1:**

It would appear my original review comments were not included in the decision letter sent to the authors after first review. I have copied these comments again here:

Response: In the first revision round, the content related review comments were mistakenly not sent to the authors. Thank you for your helpful comments. We included them into the manuscript as suggested and described below.

Summary

Authors report the results of a survey on appropriate terminology to use for describing digital mobility assessments for real world walking. Authors use the Delphi process to obtain consensus on terminology from a sample of 162 of their colleagues in the Mobilise-D research project. As acknowledged by the authors the study has limited generalisability as the sample is restricted to one research project and there is a strong risk of ‘group-think’ where students and staff reflect the view of their professors and management. However, the study is a welcome attempt to introduce a taxonomy of terms to support future research around digital mobility assessments. While the focus of the study is on terminology for real world unsupervised gait assessment, this should be placed more clearly in the context of supervised clinical and functional assessment which is the genesis of this work and will remain the reference standard against which unstructured walking will be compared. While the reports of consensus terms are welcome the findings might be more useful is they were accompanied by recommendations on their use in gait assessment studies.

Response: By using the Delphi methodology with remote and anonymous rating of the definitions, we tried to minimize the impact that senior researchers or dominant opinion leaders have on the outcome. Therefore, we aimed to minimize the risk of ‘group-think’ in the assessment and refinement (Delphi) phase of the study.

In the context of the research project (Mobilise-D), the focus of this study was, first of all, to identify and define relevant terms that need consensus with regard to unsupervised gait assessment. The developed taxonomy should be regarded as a link between supervised and unsupervised gait assessment, facilitating the transition from the first to the latter. We added respective comments, that supervised gait assessment currently is the reference standard to the discussion and the abstract.

Regarding recommendations on the use of the definitions in gait assessment studies, we tried to be general enough, such that various study settings using different sensor modalities/positions may benefit from the definitions. For walking bouts, we specifically defined that any non-gait period may terminate a bout, but did not set a specific threshold or activity. Furthermore, regarding turning, we did not aim to set specific thresholds for turning detection. More specific recommendation will be very application and sensor specific (i.e., based on whether a sensor is worn at the foot or pelvis). As stated in the discussion, the definitions are only of narrative nature. For the practical application in a system (or study), they need to be further operationalized. However, they present a solid basis and indicate which aspects to consider in real-world mobility assessment. More specific definitions of thresholds need to be determined based on real-world data evaluations and linked to clinical use cases.

**Reviewer #2:**

There are some weaknesses through the manuscript which need improvement. Therefore, the submitted manuscript cannot be accepted for publication in this form, but it has a chance of acceptance after a minor revision. My comments and suggestions are as follows:

Response: Thank you for your valuable comments. We considered and included the comments as described below.

1- Abstract gives information on the main feature of the performed study and it is focused on the background. Therefore, some details about achieved results must be added.

Response: We added information on the methods and the main results to the abstract.

2- Authors must clarify necessity of the performed research. Objectives of the study, and also differences with the previous researches must be clearly mentioned in introduction.

Response: We discuss the necessity of introducing a consented set of definitions for real-world mobility monitoring. Various studies have used different terms in ambiguous meanings. Also, there is no previous work on consenting real-world mobility assessment definitions. We highlight now the missing framework in the introduction.

3- The literature study must be enriched. In this respect, authors must read and refer to the following recent and relevant published papers: (a) wearable sensors: <https://doi.org/10.1016/j.sna.2020.112105>

(b) consensus in probabilistic: https://doi.org/10.1016/j.eswa.2020.114315

Response: Thank you for your very valuable literature suggestions.

The publication “Progress and challenges in fabrication of wearable sensors for health monitoring“ is very interesting to read. Although it touches real-world data, it focuses more on hardware and engineering aspects of measuring health related data. Therefore, we decided to not include this publication as reference.

We acknowledge the problem of setting appropriate group consensus thresholds. The suggested publication “A novel process to determine consensus thresholds and its application in probabilistic linguistic group decision-making“ is a relevant step towards the determination of thresholds. We added a paragraph in the limitation section stating the choice of our 75 % threshold and reference literature where similar thresholds have been used. However, our study does not aim to optimize group decision making processes, but rather focusses on real-world DMO assessment. Therefore, we decided to not include the reference on optimizing consensus thresholds.

4- It is strongly suggest to add figures for better description of concept and some conditions. In addition, statistical analysis is required.

Response: We added a figure about the general concept into the introduction (Fig. 1). We acknowledge that it facilitates general understanding.

Regarding the statistical analysis, we evaluated consensus based on the proportion of agreement within a range (categories "Somewhat agree" or "Strongly agree"). Similar to work which used the Delphi process to achieve consensus (e.g., Vogel et al., 2019), we decided to report only descriptive statistics (proportion of agreement / disagreement), in order to rate whether a definition has been consented. Other definitions of consensus exist (Diamond et al., 2014) and may be taken into account in similar future studies. Depending on the definition used, other statistical methods would need to be used then.

5- In its language layer, the manuscript should be considered for English language editing. There are sentences which have to be rewritten.

Response: A native English speaker thoroughly revised the manuscript in order to eliminate potential language errors.

6- The conclusion must be more than just a summary of the manuscript. List of references must be updated based on the proposed papers. Please provide all changes by red color in the revised version.

Response: In the conclusion, we restate now the research problem, summarize the main outcome and suggest key takeaways.
